# Neoadjuvant anti-PD-1 monotherapy followed by watch-and-wait strategy in dMMR/MSI-H colorectal cancer: a multicenter real-world study

**DOI:** 10.3389/fimmu.2026.1899625

**Published:** 2026-08-03

**Authors:** Tengyu Li, Huaju Zhang, Pengyu Wei, Weiyu Feng, Huiliang Zhang, Shuai Ma, Quanbo Zhou, Shuang Zhao, Luyao Shao, Haifeng Sun, Weipeng Sun, Chao Yang, Shengyun Hu, Qingqing Zhang, Xiaofei Duan, Wei Zhang, Yangbang Lian, Chang Su, Xuexiu Zhang, Zhen Li, Guixian Wang, Weitang Yuan, Zhiyong Zhang, Xuan Zhang, Hongwei Yao, Yugui Lian

**Affiliations:** 1 Department of Colorectal Surgery, The First Affiliated Hospital of Zhengzhou University, Zhengzhou, China; 2 Department of General Surgery, Beijing Friendship Hospital, Capital Medical University, Beijing, China; 3 State Key Lab of Digestive Health, Beijing Friendship Hospital, Capital Medical University, Beijing, China; 4 National Clinical Research Center for Digestive Diseases, Capital Medical University, Beijing, China; 5 Department of General Surgery, Affiliated Cancer Hospital of Zhengzhou University & Henan Cancer Hospital, Zhengzhou, China; 6 Department of Internal Medicine, Affiliated Cancer Hospital of Zhengzhou University & Henan Cancer Hospital, Zhengzhou, China; 7 The First Clinical School, Zhengzhou University, Zhengzhou, China; 8 Radiology Department, The First Affiliated Hospital of Zhengzhou University, Zhengzhou, China; 9 Department of Radiation Oncology, The First Affiliated Hospital of Zhengzhou University, Zhengzhou, China; 10 Department of Gastroenterology, The First Affiliated Hospital of Zhengzhou University, Zhengzhou, China; 11 Department of Colorectal Surgery, Yunnan Cancer Hospital & The Third Affiliated Hospital of Kunming Medical University, Kunming, China

**Keywords:** colorectal cancer, dMMR/MSI-H, endoscopic complete response, neoadjuvant immunotherapy, watch-and-wait

## Abstract

**Objective:**

This study aimed to assess the long-term results of the watch-and-wait (W&W) strategy in patients with clinical stage II/III mismatch repair deficiency (dMMR) or microsatellite instability-high (MSI-H) colorectal cancer (CRC) who received neoadjuvant anti-PD-1 monotherapy.

**Methods:**

This retrospective study enrolled stage II/III dMMR/MSI-H CRC patients from four Chinese tertiary hospitals between August 1, 2020 and February 28, 2025. All participants received neoadjuvant anti-PD-1 monotherapy followed by W&W approach. *post hoc*, endoscopic complete response (eCR) was defined as no suspicious mucosal lesion on white-light or chromoendoscopy (flat scar or normal mucosa allowed) and negative targeted biopsies from the primary tumor site. The primary outcomes were disease-free survival (DFS) and overall survival (OS). The study is registered in the Chinese Clinical Trial Registry (www.chictr.org.cn ChiCTR2500100474).

**Results:**

A total of 49 patients managed with W&W strategy were enrolled, and all met the *post hoc* eCR criteria. Radiological evaluation showed clinical complete response in 21 patients (44%), partial response in 17 (35%) and stable disease in 10 (21%). One patient lacked evaluable imaging. Median neoadjuvant treatment duration was 6 months (IQR 4-8), with a median of 12 treatment cycles (IQR 8-16). Treatment-related adverse events were grade 1–2 in 14 patients (29%); no grade 3–4 events occurred. At a median follow-up of 35 months (range 9-60), no local regrowth, distant metastasis, or death was recorded. The estimated 3-year DFS and OS are both 100%.

**Conclusion:**

In this cohort, patients with dMMR/MSI-H CRC who met eCR after neoadjuvant anti-PD-1 monotherapy had excellent long-term outcomes when managed with W&W strategy. These findings support the potential utility of endoscopic assessment for selecting candidates for organ preservation, but prospective validation of standardized criteria is warranted.

## 1 Introduction

Colorectal cancer (CRC) ranks among the most prevalent malignancies globally and is the second leading cause of cancer-related mortality worldwide (1). At the molecular level, CRC exhibits significant heterogeneity, among which microsatellite instability (MSI) serves as a pivotal molecular biomarker. Defective DNA mismatch repair (dMMR) leads to microsatellite instability-high (MSI-H) status, accounting for 10-15% of CRCs (2). These tumors are notable for a high tumor mutational burden, along with the presence of dense lymphocyte infiltration, providing the biological rationale for immune checkpoint inhibitors (ICIs) (3).

Immunotherapy has reshaped the treatment landscape of dMMR/MSI-H CRC. The KEYNOTE-177 trial established pembrolizumab as first-line therapy for metastatic dMMR/MSI-H CRC, showing superior progression-free survival and overall survival versus chemotherapy (4, 5). Subsequently, neoadjuvant immunotherapy has yielded unprecedented pathological response rates. The NICHE trials reported 68% pathological complete response (pCR) with short-course ipilimumab plus nivolumab in locally advanced dMMR colon cancer (6, 7). In rectal cancer, single-agent PD-1 blockade resulted in 100% clinical complete response (cCR), allowing omission of chemoradiotherapy and surgery (8, 9). With these high response rates, the question has shifted from “whether immunotherapy works” to “whether radical surgery can be safely omitted in responders”. The watch-and-wait (W&W) strategy, initially validated in rectal cancer after chemoradiotherapy, is now being explored after immunotherapy (10, 11). Early evidence suggests that selected dMMR/MSI-H CRC patients achieving cCR may avoid surgery without compromising oncologic outcomes (12–14). However, most evidence comes from prospective trials with strict eligibility criteria and standardized response assessment. Real-world data, especially regarding long-term outcomes and the practical performance of different response evaluation modalities, remains limited.

A critical challenge in implementing W&W strategy is the accurate identification of complete responders. Accumulating evidence reveals a substantial discrepancy between radiological imaging and pathological findings after neoadjuvant immunotherapy. For the NICHE-2 trial, only 2.7% of pCR colon cancer patients showed radiological complete remission (7). A recent meta-analysis reported an overall discrepancy rate of 59.6% between imaging and pathology, which was even higher in colon cancer (64.2%) (15). Conversely, endoscopic assessment with targeted biopsy may better reflect pathological response, as tumor regression after PD-1 blockade often follows a “mucosa-to-serosa” pattern (16). But this hypothesized tumor regression pattern is only supported by small single-institution pathological series and lacks validation in large prospective surgical cohorts.

Against this background, this multicenter retrospective real-world study aimed to report the long-term outcomes of dMMR/MSI-H CRC patients who were managed with W&W strategy after neoadjuvant anti-PD-1 monotherapy, and to describe their endoscopic findings using a *post hoc* uniform classification.

## 2 Patients and methods

This was a retrospective case series. Clinical data were collected from four high-volume CRC centers, including the First Affiliated Hospital of Zhengzhou University, Beijing Friendship Hospital (Capital Medical University), Henan Cancer Hospital, and Yunnan Cancer Hospital. All consecutive patients who received neoadjuvant monotherapy with PD-1 inhibitors between Aug 1, 2020, and Feb 28, 2025 were enrolled. We applied the inclusion filter of non-surgical management and enrolled all stage II/III MSI-H/dMMR colorectal cancer patients who received immunotherapy without radical resection across the four participating centers. *Post hoc* analysis confirmed that all enrolled patients fulfilled the predefined endoscopic complete response (eCR) criteria. The last follow-up date was Mar 31, 2026. All clinical data were collected retrospectively, but imaging and endoscopic images were subsequently reviewed centrally by independent experts blinded to patient outcomes, as detailed below.

### 2.1 Inclusion criteria

(1) diagnosis of colorectal adenocarcinoma confirmed by pathological biopsy; (2) confirmed dMMR by immunohistochemistry (loss of MLH1, MSH2, MSH6, or PMS2) or confirmed MSI-H by next-generation sequencing or polymerase chain reaction; (3) clinical stage II or III CRC (AJCC 8th edition); (4) completion of scheduled neoadjuvant anti-PD-1 monotherapy and subsequent management with W&W strategy; (5) availability of follow-up data for at least 6 months after treatment completion. Exclusion criteria included: (1) stage I or IV CRC; (2) prior radiotherapy or chemotherapy; (3) emergency surgery or previous radical resection.

There was no standardized, predefined endoscopic response criteria across participating centers. Clinical decisions regarding response evaluation, biopsy necessity, and adoption of the W&W strategy were individually determined by attending physicians at each center according to local clinical routines and after patient consultation. Clinical data were systematically retrieved from the medical files of all enrolled patients, with unified evaluation criteria defined *post hoc* as follows.

Imaging evaluation was performed to assess clinical efficacy in line with the Response Evaluation Criteria in Solid Tumors 1.1 (14). Contrast-enhanced chest-abdomen-pelvic computed tomography (CT) was applied for baseline staging of colon cancer; high-resolution pelvic magnetic resonance imaging (MRI) combined with chest-abdomen-pelvic CT was the standard staging workup for rectal cancer. Positron emission tomography-computed tomography (PET-CT) was performed only at physicians’ discretion for suspected distant metastasis, rather than a mandatory routine examination.

### 2.2 T stage criteria

All T staging followed the 8th AJCC TNM staging system. For colon cancer, CT was used to evaluate bowel wall invasion depth to distinguish T1-T4 lesions (17). For rectal cancer, MRI-based T staging was determined by evaluating muscularis propria integrity, serosal penetration and extramural venous invasion (18). Regional lymph nodes were defined as suspicious for metastasis if meeting either rule below:Short-axis diameter > 10 mm with round morphology; Short-axis diameter 5–9 mm with at least two of the following high-risk features: round shape, irregular nodal border, heterogeneous signal intensity on CT/MRI (19). Initial clinical staging was judged by attending physicians at each center. All baseline CT and MRI scans of the 49 enrolled patients underwent centralized blinded re-evaluation by two independent gastrointestinal radiologists. Any inter-reader disagreement was adjudicated by a third senior radiologist with 15 years of abdominal imaging experience. For colon tumors, the maximum axial tumor diameter on contrast-enhanced CT was defined as the target lesion dimension. For rectal lesions, the maximum tumor wall thickening measured on high-resolution pelvic MRI served as the core RECIST metric, with DWI signal loss used as an auxiliary morphological marker.

**Figure 1:**
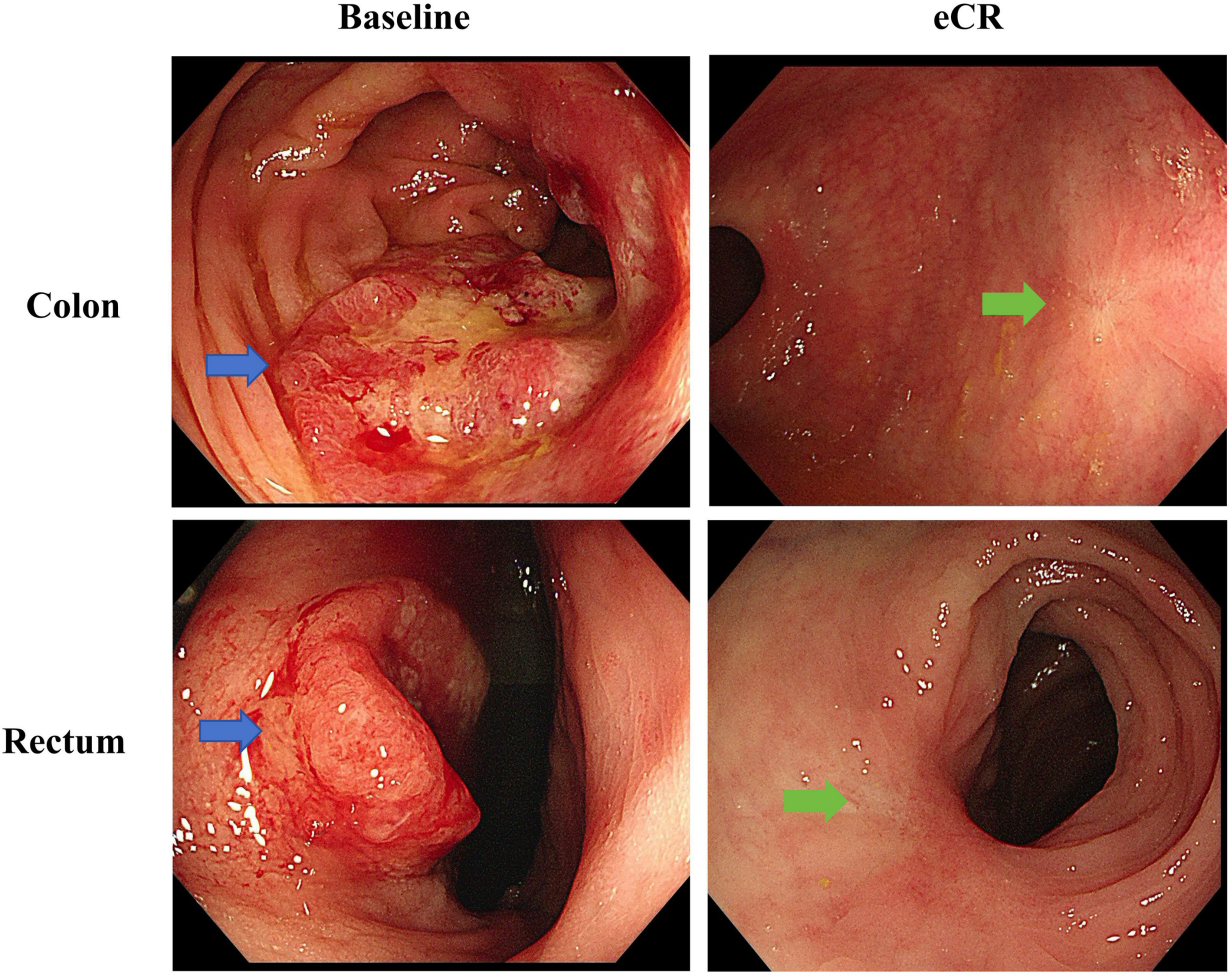
Colonoscopy images of the tumor at baseline (blue arrow) and post-treatment (green arrow).

No unified mandatory endoscopic biopsy protocol was implemented prospectively across participating centers; each institution followed its own local clinical standards during routine colonoscopy. In general, endoscopic reassessments were performed at an average interval of 3 months. At each endoscopic evaluation, at least four targeted forceps biopsies were harvested from the original primary tumor scar or flat mucosal lesion area; random non-targeted biopsies were not collected. All biopsy slides underwent centralized pathological re-review by two dedicated gastrointestinal pathologists. Endoscopic manifestations and biopsy outcomes were collected from colonoscopy reports, including mucosal conditions at the primary tumor site. To unify endoscopic response evaluation across the entire cohort, a *post hoc* eCR criterion was established for the current analysis. eCR was defined as the simultaneous satisfaction of two conditions: (1) no suspicious mucosal lesions detected on white-light or chromo-magnifying endoscopy (flat scars and normal mucosa were acceptable) (**Figure 1**); and (2) absence of viable tumor cells in targeted biopsy specimens obtained from the primary tumor site. Notably, this eCR classification was applied retrospectively for cohort outcome characterization and did not serve as a prospective clinical decision-making criterion. In addition, the timing and frequency of endoscopic examinations varied among centers and individual patients at the discretion of attending physicians. All endoscopic procedures and pathological reviews were performed by qualified specialists at each participating center in strict accordance with local clinical protocols. To ensure consistency and minimize bias, all available endoscopic images were centrally reviewed by two independent endoscopists (with 8 and 10 years of experience, respectively), who were unaware of the patients’ clinical outcomes and treatment allocation. Any disagreement was settled by consensus via a third senior reviewer. Patients underwent surveillance following the protocol described by Cercek et al. (12) Disease-free survival (DFS) was defined as the time from first anti-PD-1 administration to local regrowth, distant metastasis, death from any cause, or last follow-up. Overall survival (OS) was defined as the time from first treatment to death from any cause. The Common Terminology Criteria for Adverse Events v5.0 (20) was applied for grading all treatment-related adverse events.

Continuous data were expressed as median with interquartile range (IQR) or range, while categorical data were reported as counts and percentages. Disease-free survival (DFS) and overall survival (OS) were estimated using the Kaplan-Meier method. Follow-up time was treated as a continuous variable, with 3 years set as the predefined time point. The 3-year survival rates were interpolated from the KM survival curves. Patients with follow-up durations shorter than 36 months were treated as censored observations, while others had follow-up exceeding 36 months. All analyses were descriptive, no inferential comparisons were planned. The study protocol was approved by the Ethics Review Committee of the First Affiliated Hospital of Zhengzhou University (approval No. 2025-KY-0180-001) and by the ethics committees of each participating center. The study is registered in the Chinese Clinical Trial Registry (ChiCTR2500100474).

## 3 Results

This study enrolled 49 patients with histologically confirmed dMMR/MSI-H CRC, including 10 stage II and 39 stage III disease. All patients met the *post hoc* eCR criteria. There were 29 males and 20 females. The median age of patients was 52 years (IQR 39-63). Primary tumor sites included right colon in 18 patients (37%), left colon in 9 patients (18%), and rectum in 22 patients (45%). According to The American Society of Anesthesiologists (ASA) physical status classification, 42 patients were grade 1 and 7 were grade 2. Eastern Cooperative Oncology Group (ECOG) performance status: 0 in 31, 1 in 17, 2 in 1. Ten patients (20%) were diagnosed with Lynch syndrome; 37 had sporadic dMMR/MSI-H; germline testing results were unavailable for two (**Supplementary Table 1**).

All patients received anti-PD-1 monotherapy, including tislelizumab in 21 cases, sintilimab in 21 cases, toripalimab in 4 cases, camrelizumab in 1 case, and pembrolizumab in 1 case. One patient received sequential tislelizumab and camrelizumab. Median treatment duration was 6 months (IQR 4-8), equivalent to 12 cycles (IQR 8-16). (**Supplementary Table 1**) Fourteen patients (29%) experienced grade 1–2 treatment-related adverse events (AEs), including pruritus, nausea, elevated transaminases, hypothyroidism, and other manifestations. No grade 3–4 AEs occurred (**Table 1**).

**Table 1:** Summary of immune-related adverse events in enrolled patients.

	All grades no. patients (%)	Grade 3 or 4 no. patients (%)
Dermatologic		
Pruritus	2(4)	0
Gastrointestinal		
Nausea	1(2)	0
High ALT/AST	4(8)	0
High GT	4(8)	0
Endocrine		
Hypothyroidism	4(8)	0
High ACTH	1(2)	0
Circulatory		
Myocarditis	1(2)	0
Other		
High LDH	2(4)	0
Low Uric Acid	1(2)	0

Alanine aminotransferase, ALT; Aspartate aminotransferase, AST; glutamyl transpeptidase, GT; adrenocor ticotropic hormore, ACTH; Lactate dehydrogenase, LD.H.

All 49 patients underwent serial endoscopic examinations during neoadjuvant therapy and subsequent surveillance. The median number of endoscopic examinations was 2 (IQR, 2–3), with 2 examinations in 25 patients (51%), 3 examinations in 22 patients (45%), and ≥ 4 examinations in 2 patients (4%).

**Figure 2:**
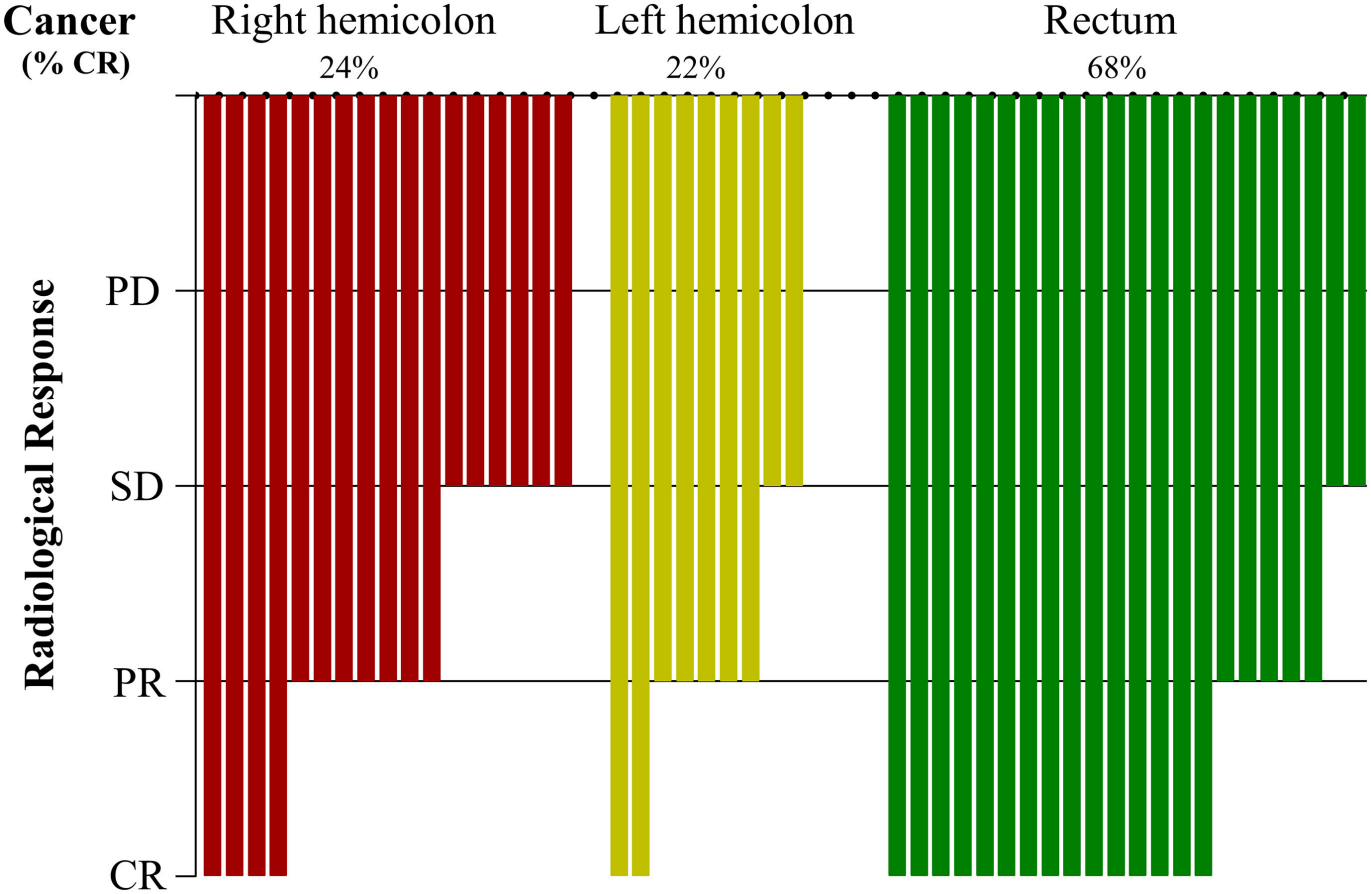
Radiological response by tumor location. Tumor responses are defined as clinical complete response (CR), partial response (PR), stable disease (SD) and Progressive disease (PD).

**Figure 3:**
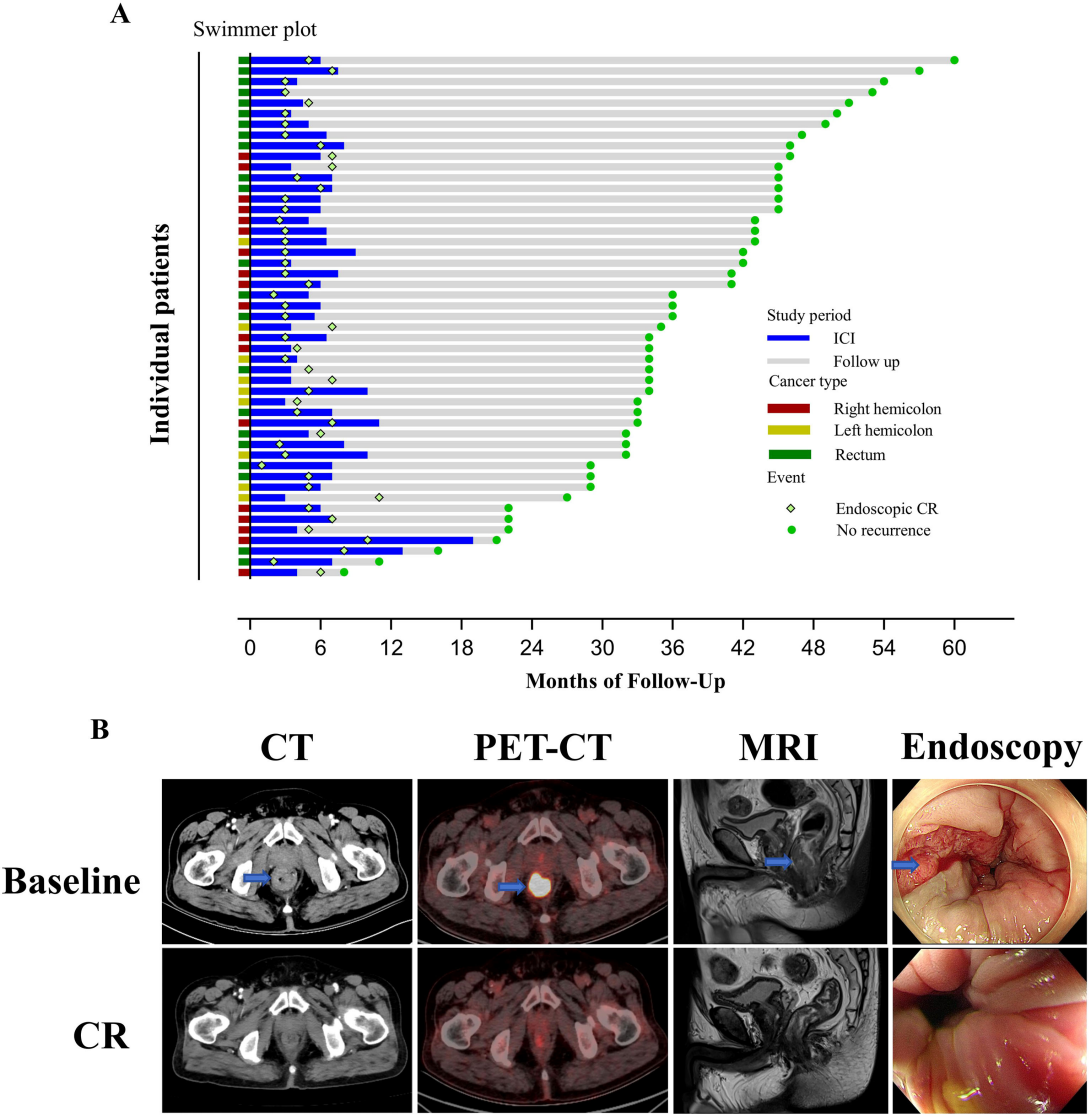
**(A)** Swimmer plot of each patient’s response and duration. **(B)** Clinical Vignette. A 54-year-old male with a localized dMMR rectal adenocarcinoma. The lower edge of the tumor is 0 cm from the anus. The above images show the CT, PET-CT, MRI, and colonoscopy images of the tumor at baseline (blue arrow). The images below show complete remission on CT, PET-CT, MRI, and colonoscopy after 19 cycles of immunotherapy treatments.

Based on imaging, the best overall response was: complete response (cCR) in 21 patients (44%, 95% CI: 30-58), partial response (PR) in 17 (35%, 95% CI: 23-50), and stable disease (SD) in 10 (21%, 95% CI: 10-35). One patient lacked evaluable imaging. Notably, although only 21 patients (44%) had radiological cCR, the discrepancy rate between radiological cCR and eCR was 56% (27/48 patients had non-cCR on imaging but met eCR criteria). When stratified by tumor location, the discordance rate was 77% (20/26) for colon cancer and 32% (7/22) for rectal cancer. Radiological response of tumors at different anatomical sites was shown in the waterfall plot (**Figure 2**), and individual patient timelines were presented in the swimmer plot (**Figure 3**).

**Figure 4:**
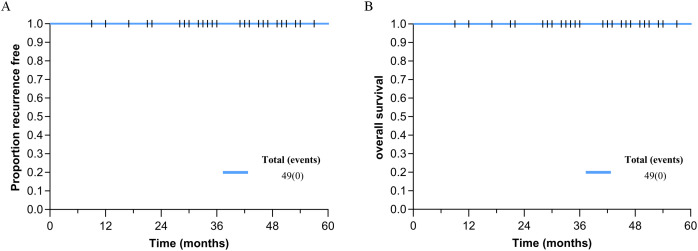
**(A)** Time-to-recurrence was defined as the interval from the initiation of PD-1 inhibitor therapy to tumor recurrence. **(B)** Kaplan-Meier survival curves for overall survival.

At a median follow-up of 35 months (range 9-60), no patient developed local regrowth, distant metastasis, or died. The estimated 3-year DFS and OS are both 100% (**Figure 4**).

## 4 Discussion

This multicenter retrospective study of 49 dMMR/MSI-H CRC patients treated with neoadjuvant anti-PD-1 monotherapy and subsequently managed with W&W strategy yielded three main findings. First, the regimen was associated with 100% 3-year DFS and OS. These exploratory real-world observations suggest favorable long-term oncologic outcomes within this highly selected retrospective W&W cohort. Second, a marked discordance was noted between radiological and endoscopic assessment, 44% radiological complete response vs. 100% meeting *post hoc* endoscopic complete response (eCR) criteria, which may reflect limited specificity of radiological evaluation after neoadjuvant immunotherapy. Third, no salvage surgery was required during follow-up among patients achieving eCR, suggesting that this state may be a useful indicator for successful organ preservation.

The high response rate observed in our cohort aligns with prospective trials. The NICHE-2 trial reported 68% pCR after short-course dual immunotherapy (13). The IMHOTEP trial showed 52.7% pCR after 1–2 cycles of pembrolizumab (21). RESET-C trial demonstrated 44% pCR with a single cycle of pembrolizumab in colon cancer (22). However, although our 100% eCR rate appears higher, direct comparison is limited by different endpoints (pCR vs. eCR) and the fact that eCR, defined by mucosal healing with negative biopsy, may overestimate pCR due to inability to assess deeper wall or nodal residuals. Nevertheless, the 100% 3-year DFS and OS supports the oncologic safety of this clinical practice.

This study demonstrated a favorable safety profile, with only grade 1–2 adverse events (AEs) observed in 29% of patients and no grade 3–4 AEs recorded. This compares favorably with dual ICI regimens (4-22% grade 3-4 irAEs) (13, 23) and anti-angiogenic combinations (38% grade 3-4 irAEs) (24). Considering that real-world patients generally have more complex clinical profiles than those recruited in clinical trials, the favorable tolerability observed in this cohort supports single-agent PD-1 blockade as a well-tolerated neoadjuvant option.

One of the intriguing findings is that only 44% of patients achieved radiological cCR, yet all W&W patients had documented eCR. This discordance is consistent with emerging evidence. In the NICHE-2 trial, only 2.7% of pCR colon cancer patients showed radiological complete remission (13). A meta-analysis by Xie et al. including 396 patients reported an overall imaging-pathology discordance rate of 59.6%, with colon cancer showing even higher discordance (64.2%) than rectal cancer (34.9%) (15). Fox et al. found that 72% of patients with clinical benefit still had non-CR on imaging (24). The mechanisms underlying this phenomenon include post-immunotherapy fibrosis, immune cell infiltration, and acellular mucin pools, which can mimic residual tumor on CT/MRI (25). RECIST 1.1 depends only on lesion dimensions and fails to differentiate intralesional fibrosis, acellular mucin pools from viable neoplastic tissue, which results in radiological underestimation of pCR (26). Therefore, relying solely on radiological criteria to select patients for W&W strategy would likely lead to unnecessary surgery in a large proportion of true responders.

Our data showed that patients who met eCR had excellent outcomes with W&W management. This supports endoscopic assessment as a useful tool for selecting candidates for organ preservation. Because the response pattern after PD-1 blockade often follows a “mucosa-to-serosa” direction, negative mucosal biopsies may indicate the absence of residual invasive disease at the primary site (16). However, this is a retrospective observation; biopsy decisions and interpretations were not standardized across centers. Negative targeted mucosal biopsies may only suggest low risk of superficial residual lesions on the luminal surface, yet cannot fully exclude microscopic residual tumor within deep bowel wall layers or regional lymph nodes.

At present, there is no widely accepted or standardized definition of cCR for dMMR/MSI-H CRC after neoadjuvant immunotherapy. Most published studies have used varying combinations of radiology, endoscopy, and biopsy, making cross-trial comparisons difficult. Recently, Liao et al. proposed an immune-heralding complete response (iHCR) model for dMMR colon cancer, requiring at least two of three criteria: endoscopic normal mucosa/scar, negative biopsy, and ≥50% radiological tumor reduction. In their cohort with complete endoscopic and radiological data, the iHCR model achieved an AUC of 0.900 (95% CI 0.810–0.990), with 100% specificity and 80% sensitivity; external validation in the NEOCAP trial yielded an AUC of 1.00. This model accurately predicted pCR, and all 32 patients who met iHCR criteria remained recurrence-free under W&W management (27). Although our eCR definition was developed retrospectively only for cohort description, we compared it with the iHCR model to explore differences in stringency. Our eCR definition only adopts the first two criteria, excluding radiological shrinkage, for three key reasons. First, radiological response lags behind pathological response after immunotherapy. In our cohort, only 44% of eCR patients had radiological complete response, adding this criterion would unnecessarily exclude more than half of true responders from organ preservation. Second, the iHCR model was validated mainly in surgically treated cohorts, lacking prospective evidence for W&W decision-making, whereas our eCR definition is validated by real-world long-term outcomes. Third, the two-criterion eCR is more clinically practical, as endoscopic assessment with biopsy is widely available, low-cost, and less prone to inter-observer variability than radiological measurements. Admittedly, our eCR definition has limitations. The iHCR model’s radiological criterion may offer additional safety for high-risk subgroups, such as mucinous tumors, bulky T4b lesions, or node-positive disease. Our cohort included few such patients, so the necessity of radiological criteria for these subgroups cannot be excluded.

Unlike prospective trials that predefined cCR with radiological thresholds, this real-world study used a *post hoc* two-criterion definition. We found that all long-term survivors met this definition, even though half of them never achieved radiological CR. Endoscopic evaluation combined with targeted biopsy shows an exploratory correlation with favorable survival outcomes in this cohort; however, standardized eCR criteria must undergo prospective validation before being considered for routine screening of organ-preservation candidates. These findings do not replace prospective validation, but they provide a real-world benchmark for future efforts to standardize cCR criteria. Importantly, the 100% eCR rate observed here should not be interpreted as “all treated patients achieved eCR” but rather as “among those who were managed with W&W management and had documented endoscopic and biopsy data, all met *post hoc* eCR definition”. It is critical to reiterate that the eCR definition adopted in this study was established *post hoc* for consistent cohort characterization; it was not implemented as a prospective criterion for treatment discontinuation across participating centers. While our observation that all patients meeting *post hoc* eCR criteria remained recurrence-free over a median follow-up of 35 months suggests that endoscopic remission may serve as a valuable indicator to identify candidates for organ preservation, this finding does not confirm that eCR can or should be adopted as the sole threshold for treatment discontinuation in routine clinical practice. Endoscopic mucosal healing does not always equate to complete eradication of tumor within the deep bowel wall or mesorectal lymph nodes. Xie et al. reported that 10%-18% of patients with isolated endoscopic remission still harbored residual viable tumor cells within surgical specimens (15). The absence of disease recurrence within our cohort may partially reflect the late tail effect of immunotherapy. However, the absence of standardized treatment discontinuation protocols and control comparator subgroups prevents drawing any causal inference that early treatment cessation following mucosal remission is oncologically safe. Prospective clinical trials are warranted to clarify whether eCR either alone or combined with radiological and molecular biomarkers can be established as a validated stopping criterion for routine clinical use.

Our median treatment duration of 6 months (12 cycles) was longer than that in the NICHE series (1–2 cycles) but similar to the dostarlimab regimen (13). The optimal treatment duration remains undefined. The IMHOTEP trial showed a dose-effect relationship: pCR increased from 46% after 1 cycle to 68% after 2 cycles of pembrolizumab (21). A systematic analysis by Rousseau et al. reported that longer treatment duration was correlated with a higher complete response rate (28). In our real-world cohort, patients received varying durations (4-8 months) based on physician and patient preference, yet all met eCR. This suggests that a shorter course might suffice, but our data cannot definitively answer this question. Ongoing trials, including AZUR-2 (NCT05855200), NEOSHOT (NCT05890742), and OPEN (NCT06698757), are investigating optimal treatment duration and drug combination strategy (29, 30).

Several limitations should be acknowledged. The retrospective, non-comparative design introduces inherent selection bias, and the relatively small sample size limits statistical power. Treatment heterogeneity arose from the use of multiple PD-1 inhibitors, which reflects real-world practice but complicates interpretation. Although central blinded review of imaging and endoscopy was performed, the assessment of response was still based on retrospective data collection. While the median follow-up (35 months) exceeds that of most published studies, late recurrences beyond three years cannot be excluded. Finally, the favorable outcomes may partly reflect patient selection (relatively young, median age 52, and good performance status), and caution is warranted when extrapolating these results to older or frailer populations. Moreover, the eCR criteria were defined *post hoc* and have not been prospectively validated. Therefore, while the findings are encouraging, they should be considered hypothesis-generating rather than practice-changing at this stage. Despite the limitations, our findings have practical implications. For dMMR/MSI-H CRC patients who, after neoadjuvant anti-PD-1 monotherapy, are found to have no visible mucosal lesion and negative targeted biopsy, immediate surgery may be safely deferred in favor of W&W strategy with close surveillance. Radiologic non-CR should not automatically disqualify patients from W&W strategy if eCR criteria are met. Future prospective studies should standardize eCR criteria, incorporate circulating tumor DNA monitoring and functional imaging to create a multimodal response assessment algorithm, and define the minimal effective treatment duration.

## 5 Conclusion

In this multicenter cohort, patients with stage II/III dMMR/MSI-H CRC who met the *post hoc* eCR after neoadjuvant anti-PD-1 monotherapy had excellent 3-year DFS and OS when managed with W&W strategy. While these findings suggest that endoscopic assessment may help identify suitable candidates for organ preservation, the retrospective design precludes definitive conclusions. Prospective validation of standardized endoscopic response criteria is warranted.

## Data Availability

The raw data supporting the conclusions of this article will be made available by the authors, without undue reservation.
